# Subungual Amelanotic Melanoma Masquerading as Onychomycosis

**DOI:** 10.7759/cureus.2307

**Published:** 2018-03-11

**Authors:** Ryan R Riahi, Philip R Cohen, Leonard H Goldberg

**Affiliations:** 1 Dermatology, Derm Surgery Associates, PA; 2 Department of Dermatology, University of California, San Diego

**Keywords:** amelanotic, malignant, masquerading, melanoma, mimicking, nail, onychomycosis, subungual, tumor, unguium

## Abstract

Subungual amelanotic melanoma is rare. In addition, amelanotic melanoma can mimic non-melanocytic tumors. A 67-year-old woman had a four-year history of dystrophy of the left fourth fingernail. Periodic acid-Schiff staining of the nail plate demonstrated fungal hyphae, establishing a diagnosis of tinea unguium. The nail plate subsequently detached and the underlying nail bed showed a red, friable mass that was biopsied and confirmed a diagnosis of melanoma. In conclusion, additional morphologic change of a persistent nail dystrophy—even with a biopsy-confirmed diagnosis of onychomycosis—may require consideration for repeat evaluation, including a biopsy, to exclude the possibility of a subungual malignant tumor.

## Introduction

Subungual amelanotic melanoma can be a diagnostic challenge both clinically and pathologically. Subungual melanomas represent approximately two percent of melanomas; of these, 15% to 25% are amelanotic. Subungual melanomas most commonly involve the nails of the great toes and thumbs [1-2].

Amelanotic melanoma—whether cutaneous or subungual—can mimic non-melanocytic lesions [3-7]. Indeed, it typically presents as a red nodule. Hence, subungual amelanotic melanoma is associated with a poor prognosis due to misdiagnosis, delay in diagnosis, or both [1,4].

The features of a 67-year-old woman who presented for the evaluation of fingernail dystrophy of the left fourth digit are described. Although a periodic acid-Schiff staining of nail clippings confirmed the presence of fungal hyphae, a subungual amelanotic melanoma was discovered after the dystrophic nail plate spontaneously detached. The differential diagnosis of red subungual tumors is summarized and the importance of considering additional etiologies for the diagnosis of an established, yet progressive, nail dystrophy—one that not only persists but changes in morphology—is emphasized.

## Case presentation

A 67-year-old woman presented for the evaluation of a fingernail dystrophy of the left fourth digit of four years' duration. She noted that the dystrophy appeared after a manicure. The dystrophic nail had previously been avulsed and subsequently grown back; however, the dystrophy persisted.

Evaluation of the left fourth digit of the hand demonstrated a nail with excessive ridging and prominent subungual debris. There was neither elevation of the nail plate nor pigmentation of the proximal nail fold. There were no other dystrophic fingernails or toenails. The differential diagnosis included onychomycosis and trauma-induced nail dystrophy.

**Figure 1:**
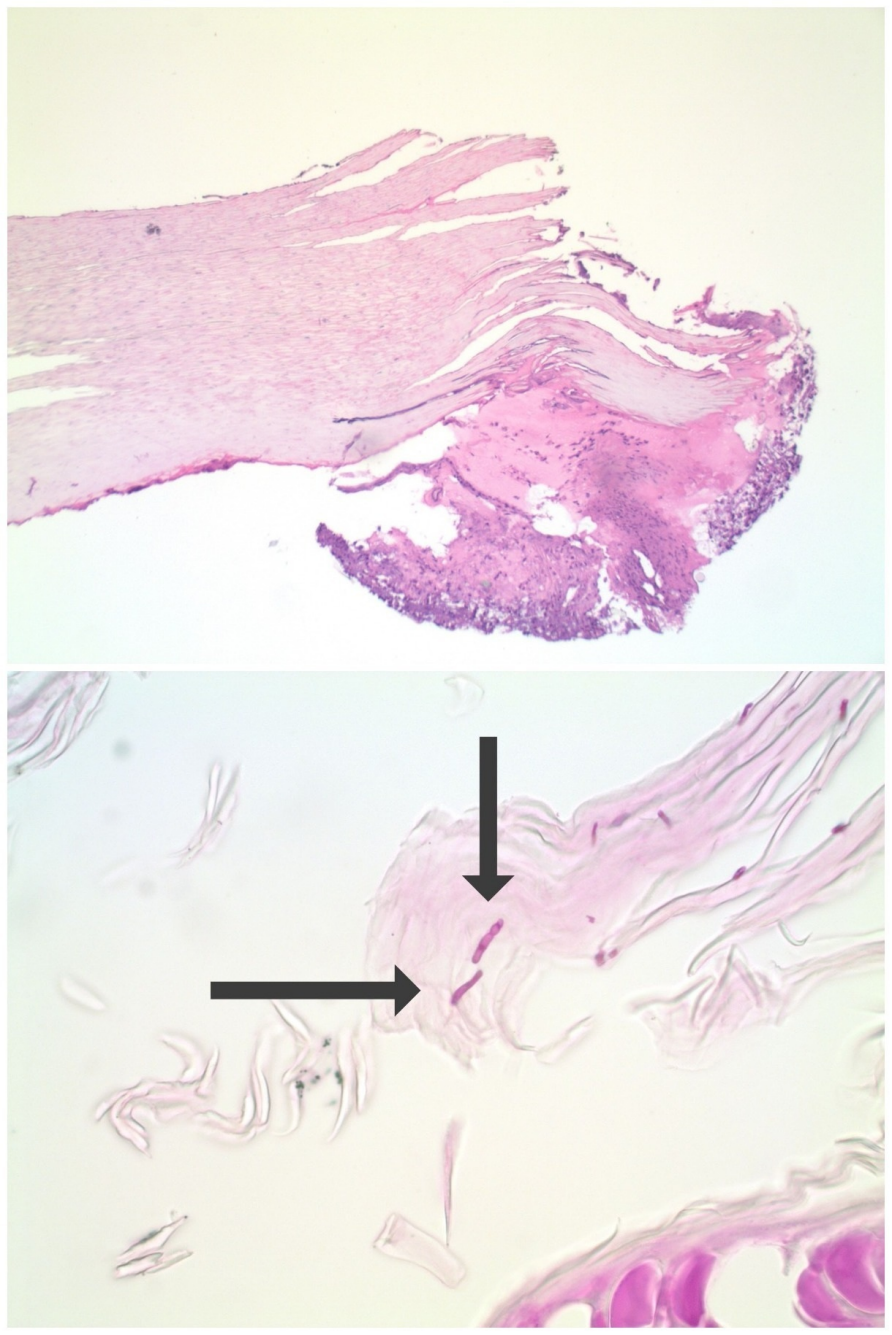
Onychomycosis demonstrated by periodic acid-Schiff staining of fingernail clippings. Distant (top) and closer (bottom) views of the fingernail clippings from the left fourth digit dystrophic fingernail of a 67-year-old woman who was submitted for microscopic evaluation; periodic acid-Schiff staining shows purple-stained hyphae (arrows), establishing the diagnosis of tinea unguim (Periodic acid-Schiff: a, x2; b, x40).

Nail clippings were obtained; periodic acid-Schiff staining demonstrated fungal hyphae within the nail plate (Figure 1). The diagnosis of onychomycosis was established and considered to be the cause of her nail dystrophy. She declined oral antifungal therapy.

**Figure 2:**
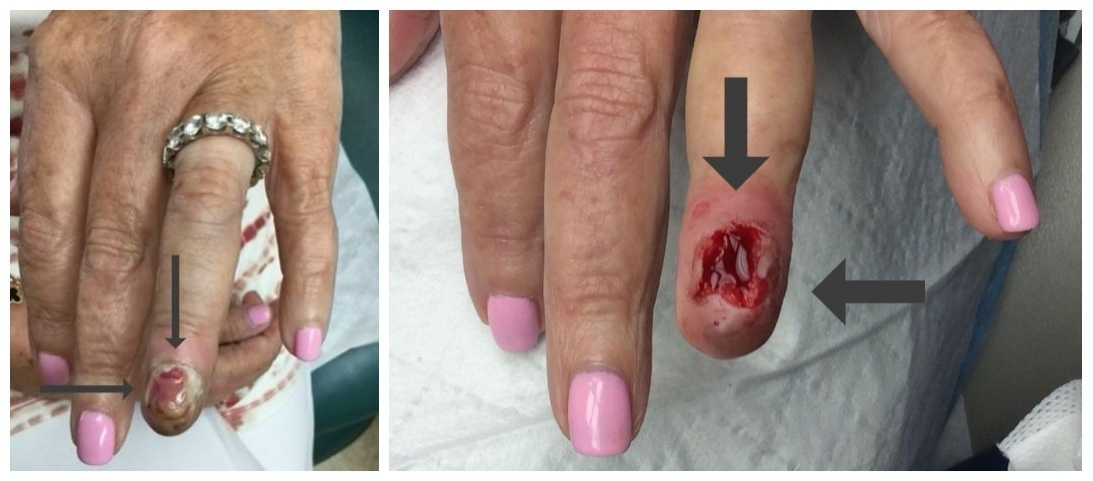
Amelanotic subungual melanoma presenting as a red nodule and the site following biopsy. The left fourth digit of a 67-year-old woman’s hand (left) shows the loss of the fingernail with an underlying red friable nodule (long, thin arrows). An erythematous, bleeding nail bed (right, thick arrows) is noted on the distal left fourth digit of a 67-year-old woman’s hand after a shave biopsy of the subungual nodule was performed (right).

She presented for follow-up three months later; her left fourth fingernail had spontaneously detached. Examination revealed a friable, erythematous nodule (Figure 2). The differential diagnosis included pyogenic granuloma and squamous cell carcinoma. A shave biopsy was performed (Figure 2).

**Figure 3:**
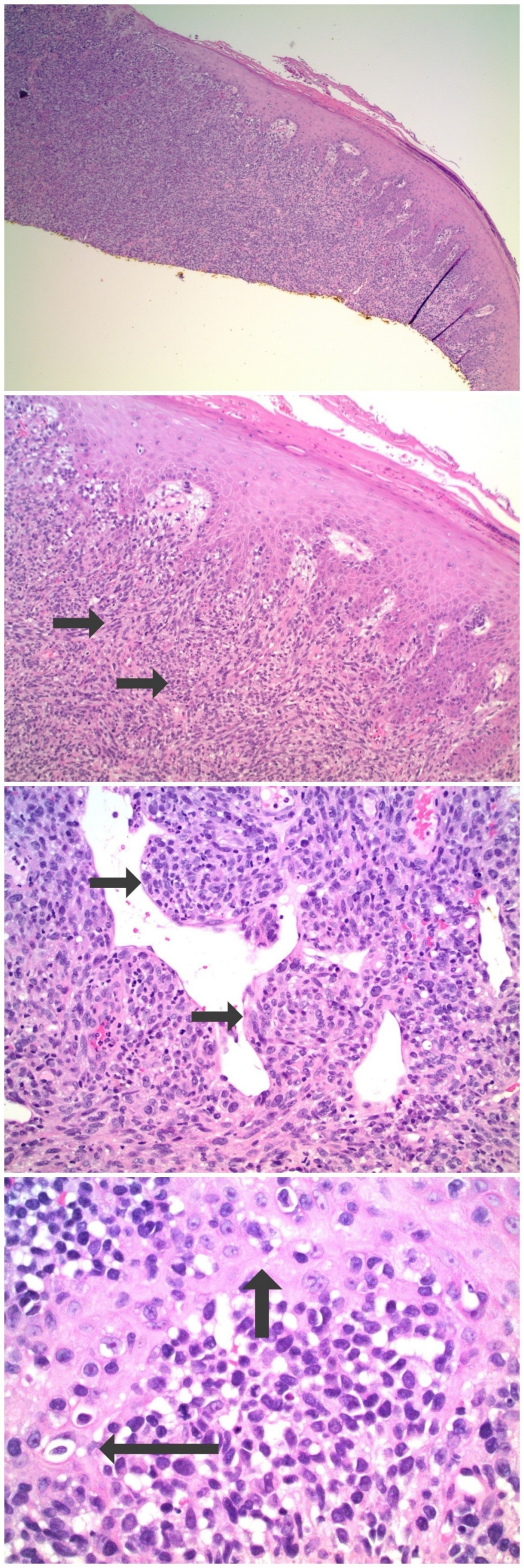
Microscopic examination of the hematoxylin and eosin stained nail bed biopsy from a subungual amelanotic melanoma. Distant (top) and closer (lower) views show nests of malignant tumor cells that fill the dermis (short arrows). The neoplasm does not demonstrate any pigment. Individual tumor cells extend into the overlying epidermis (longer arrows in the bottom image) (Hematoxylin and eosin: top x4; middle upper x10; middle lower x10; bottom x40).

**Figure 4:**
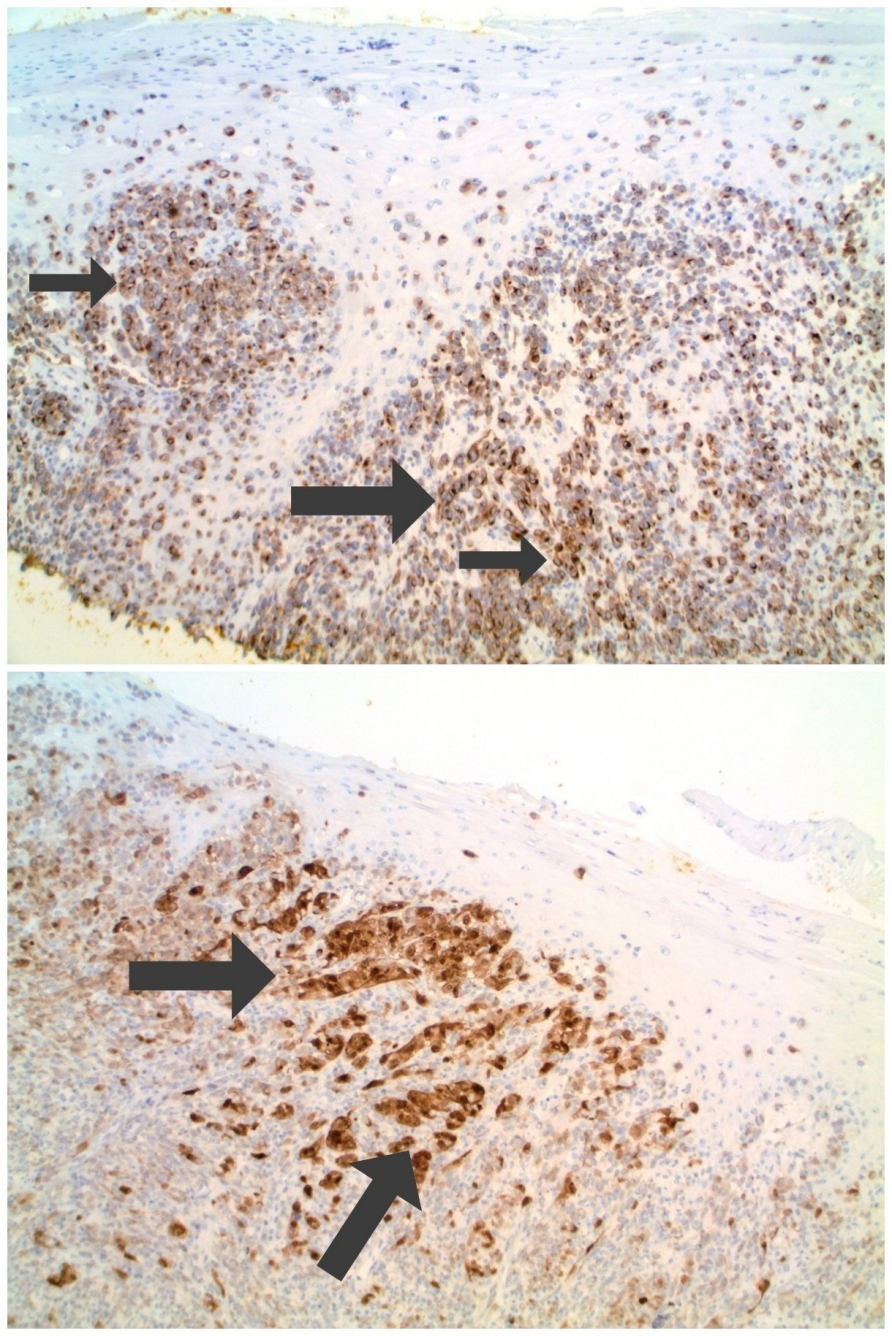
Microscopic examination of the melanoma-associated antigen recognized by T cells 1 (MART-1) and S100 immunoperoxidase stained nail bed biopsy from a subungual amelanotic melanoma. There is brown staining (arrows) of the subungual amelanotic melanoma tumor cells confirming melanocyte lineage (Melanoma-associated antigen recognized by T cells 1 immunoperoxidase: top x10; S100 immunoperoxidase, bottom x20). The bottom view demonstrates epidermis and underlying dermis; there is brown staining (arrows) of the subungual amelanotic melanoma tumor cells in the dermis, after staining with S100, which confirms melanocyte lineage.

Microscopic examination of the biopsy specimen showed a tumor that was not only present in the epidermis (which also had ulceration) but also filled the dermis and extended to the base of the biopsy at a depth of 1.9 millimeters (Figure 3). The tumor consisted of large irregular nests of non-pigmented, cytologically atypical cells; melanoma-associated antigen recognized by T cells 1 (MART-1) and S100 staining of the cells confirmed that they were melanocytes (Figure 4). Hence, the neoplasm was an amelanotic melanoma.

A correlation of the clinical presentation and pathology findings established the diagnosis of a subungual amelanotic melanoma. The patient was referred to a surgical oncologist for further evaluation and management. Systemic work-up did not demonstrate metastatic disease. A sentinel lymph node biopsy from the left axilla was negative for melanoma. She subsequently underwent an amputation of the digit. She is currently disease-free and regularly receives total body skin checks and follow-up evaluations by the surgeon.

## Discussion

Melanoma may be suspected when a pigmented lesion has a black, blue, or brown color. Melanoma types include acral lentiginous, desmoplastic, lentigo maligna, melanoma in-situ, mucosal, nodular, and superficial spreading. Subungual melanoma is a rare acral lentiginous melanoma subtype that may present as an amelanotic tumor without pigment [2].

A subungual amelanotic melanoma typically presents as a non-pigmented erythematous nodule arising from the nail bed. However, the differential diagnosis of a subungual red lesion is broad (Table 1) [1-3,5-10]. Therefore, a delay in the diagnosis of a subungual amelanotic melanoma can occur due to its morphologic resemblance to other benign and malignant neoplasms or nail conditions, such as lichen planus and onychomycosis [4].

**Table 1:** Differential diagnosis of red subungual lesions. ^1^Squamous cell carcinoma includes invasive tumor, in situ carcinoma, and keratoacanthoma.

Differential diagnosis	Reference
Callus	[6]
Foreign body reaction	[6]
Hematoma	[6]
Infection: bacterial	[6]
Infection: deep fungal	[1]
Melanoma: amelanotic	[1,2,5-10]
Metastasis: cutaneous	[3]
Pyogenic granuloma	[5]
Sarcoidosis	[1]
Squamous cell carcinoma^1^	[1,6]
Ulceration	[6,10]
Verruca vulgaris	[6]

In our patient, onychomycosis was initially considered. Nail clippings demonstrating a periodic acid-Schiff-positive staining of hyphae confirmed the diagnosis. However, the concurrent diagnosis of subungual amelanotic melanoma was established when the patient presented for follow-up and a biopsy was performed to evaluate the friable, red nodule that was noted after her nail plate had spontaneously detached.

Patients presenting with new and/or solitary nail dystrophy warrant evaluation. A complete examination of the patient’s skin and nails is recommended; a solitary nail dystrophy could be the initial presentation of a subungual lesion beneath the nail plate. Nail clippings for the microscopic examination of hematoxylin and eosin- and periodic acid-Schiff-stained sections can be performed to evaluate for onychomycosis. Nail avulsion for further evaluation, with possible biopsy of the underlying nail bed, should be performed if a neoplasm is suspected.

Persistent or progressive nail dystrophy, even with a previous, biopsy-confirmed diagnosis may require consideration for additional evaluation or biopsy or both. Our patient’s dystrophic nail had previously been removed with unremarkable findings. We initially obtained nail clippings of the dystrophic nail and demonstrated hyphae, confirming that fungus was present in the nail plate. However, the concurrent diagnosis of subungual amelanotic melanoma was not established until her nail detached and a biopsy of the previously unsuspected nail bed tumor was performed. Therefore, in individuals with a new and/or solitary nail dystrophy and in patients with a persistent or progressive nail dystrophy, the clinician should maintain a high index of suspicion for an associated condition or tumor and ensure regular follow-up.

## Conclusions

Subungual amelanotic melanoma is rare. Patients presenting with nail dystrophy—particularly if the dystrophy involves a single nail— should be thoroughly evaluated and an underlying neoplasm or condition as the etiology of the dystrophy should be entertained; scheduled follow-up for evaluation and possible additional testing is recommended for individuals with a persistent nail dystrophy. Subungual amelanotic melanoma has the potential to mimic other benign and malignant conditions; therefore, it should be considered in any patient with a solitary nail dystrophy, even with an established diagnosis, who does not respond to appropriate therapy.
